# Aging impacts memory for perceptual, but not narrative, event details

**DOI:** 10.1101/lm.053740.122

**Published:** 2023-02

**Authors:** Angelique I. Delarazan, Charan Ranganath, Zachariah M. Reagh

**Affiliations:** 1 Department of Psychological and Brain Sciences, Washington University in St. Louis, St. Louis, Missouri 63130, USA; 2 Center for Neuroscience, University of California, Davis, Davis, California 95618, USA

## Abstract

Memory is well known to decline over the course of healthy aging. However, memory is not a monolith and draws from different kinds of representations. Historically, much of our understanding of age-related memory decline stems from recognition of isolated studied items. In contrast, real-life events are often remembered as narratives, and this kind of information is generally missed in typical recognition memory studies. Here, we designed a task to tax mnemonic discrimination of event details, directly contrasting perceptual and narrative memory. Older and younger adults watched an episode of a television show and later completed an old/new recognition test featuring targets, novel foils, and similar lures in narrative and perceptual domains. While we observed no age-related differences on basic recognition of repeated targets and novel foils, older adults showed a deficit in correctly rejecting perceptual, but not narrative, lures. These findings provide insight into the vulnerability of different memory domains in aging and may be useful in characterizing individuals at risk for pathological cognitive decline.

Memory decline is among the most commonly reported cognitive changes with aging (Craik 1994; Bäckman et al. 2001; Salthouse 2003). In particular, older adults appear to show marked decline in the ability to support episodic memories for specific events and instances (Nilsson 2003; Salthouse 2003; Hedden and Gabrieli 2004). Older adults reliably show deficits when freely recalling studied information (Craik and McDowd 1987; Gutchess et al. 2006) or remembering specific item–context pairings (Old and Naveh-Benjamin 2008; Craik et al. 2010). In contrast, older adults do not consistently show deficiencies in old/new recognition memory. This and related evidence have led to the view that older adults have preserved memory for gist, but loss of specific details (Schacter et al. 1997; Abadie et al. 2021; Grilli and Sheldon 2022). That is, older adults tend to remember a general understanding of the overall experience but are disadvantaged at maintaining precise, high-fidelity details (Radvansky et al. 2001). However, it is not well understood whether such relative loss of detailed memory extends across information domains.

The Mnemonic Similarity Task (MST) is a recognition paradigm that is specifically designed to tax high-fidelity memory representations (Kirwan and Stark 2007; Stark et al. 2013, 2019). MST performance depends on maintaining similar representations in memory as distinct and nonoverlapping (Yassa and Stark 2011). This is thought to rely on pattern separation in the hippocampus (McClelland et al. 1995; Norman and O'Reilly 2003; Leutgeb et al. 2007; Bakker et al. 2008), a process that is strongly impacted in the aging brain (Wilson et al. 2006; Burke et al. 2010). Typical MST paradigms involve an incidental encoding task, such as making indoor or outdoor judgments for pictures of everyday objects, and then a surprise recognition memory test. In the memory test, participants are tasked to identify exact repetitions of previously encoded objects (targets), new objects (foils), and objects that are perceptually similar to images encountered during the encoding task (lures) as old or new. Older adults are more likely to endorse similar lures as previously studied items (Toner et al. 2009; Holden et al. 2013), which correlates with aberrant structural and functional properties of the human hippocampus and surrounding cortical regions (Yassa et al. 2011a; Reagh et al. 2018). The MST therefore offers mechanistic insights into high-fidelity recognition-based memory in the human brain.

Nonetheless, studies using the MST have often limited their scope to detecting visual changes among isolated items. Other recognition studies that have incorporated discrimination of highly similar information in more complex formats, such as source memory discrimination, also report age-related deficits (Schacter et al. 1991; Chalfonte and Johnson 1996; Naveh-Benjamin et al. 2003; Dennis et al. 2008). For instance, older adults had difficulty assessing the source of a word when it originated from two female speakers compared with across gender speakers (Ferguson et al. 1992). These studies, however, largely assess recognition memory processes for isolated items—snapshots of perceptual experience in the context of a laboratory experiment. Moreover, there is growing evidence that aging does not equally impact all domains of information that are involved in constructing a memory. For instance, recent work suggests that aging distinctly influences medial temporal lobe circuits underlying memory for items versus contexts or space (Reagh et al. 2016, 2018; Berron et al. 2018).

Real-world memories are not made of isolated pieces of information, but instead are structured and bridged together by meaning (Schank 1975; Conway and Rubin 2019; Cohn-Sheehy et al. 2022). Prior studies have shown that older adults are relatively impaired at detecting and remembering perceptual changes in everyday events, suggesting that basic findings from MST paradigms likely translate to real-world deficits (Wahlheim and Zacks 2019). However, a critical component of human memory is information about narratives, whether autobiographical or fictional (Radvansky et al. 2005; León 2016). Narratives tend to be organized to follow an ideal internal structure that can be relied on (Mandler and Johnson 1977; Thorndyke 1977). Studies that use narratives to test memory typically task participants with recalling information from a story or event. This has led to the idea that aging impacts recall more drastically than recognition (Danckert and Craik 2013). Interestingly, similar to studies of recognition memory, recall performance in aging has been characterized by loss of specific details and emphasis on information that capture the central idea of an experience (Addis et al. 2008). This may be due to the unconstrained nature of recall tasks or because self-initiated recall may be more taxing for older adults. Thus, the extent to which narrative details are truly lost versus not voluntarily retrieved in aging remains unclear. In line with this idea, tasks designed to drive participants to recall events in terms of specific details have shown enhancement effects in older adults (Madore et al. 2014). To our knowledge, however, there has not been an investigation into whether recognition of highly specific narrative details is affected in aging similarly to perceptual details.

Testing of narrative and perceptual domains alongside one another in a controlled and highly similar way allows us to gain a better understanding into the processing of different types of information in memory. Memory is not a unitary phenomenon, and memory performance can often be based on multiple processes and types of representations. This approach offers unique insights into the aging brain, as it has been previously proposed that information about narratives and situations may be preferentially encoded differently in distinct cortical pathways to the hippocampus compared with more perceptually focused information (Ranganath and Ritchey 2012; Reagh and Ranganath 2018). Other emerging neural evidence suggests that specific networks specialize in cognitive processes that are relevant for gist and detailed memory (Robin and Moscovitch 2017; Sekeres et al. 2018). Given that these brain networks may be distinctly vulnerable to age-related pathologies (Jagust 2018; Maass et al. 2019), these insights may further offer us clues into pathological aging.

Here, we designed a task to simultaneously tax mnemonic discrimination in perceptual and narrative domains. This task is analogous to traditional MST paradigms composed of an incidental encoding task followed by a recognition test. However, with the goal of tapping into mechanisms involved in encoding of the meaningful, continuous, and dynamic world that we live in, the incidental encoding task consists of watching a television sitcom (HBO's *Curb Your Enthusiasm*, S01E07: “AAMCO”) (see Fig. 1A). Television shows offer a unique methodology that balances realistic scenarios while directing our attention to specific perceptual and narrative details. After encoding, participants completed an old/new recognition test featuring targets, foils, and similar lures in the perceptual domain, as well as a novel variant testing mnemonic discrimination of narrative details. This allowed us to test detailed memory for perceptual and narrative information using an ecologically valid yet constrained approach. That is, encoding involves an immersive stimulus that hinges on meaningful and nonarbitrary narrative organization. Additionally, although retrieval is akin to a standard recognition test, it assesses memory along two dimensions that may provide insight into how we process different memory representations for lifelike events. Unlike prior studies testing narrative understanding, here we critically tested narrative memory in terms of basic recognition (targets and foils) as well as high-fidelity narrative details (lures). Performance was compared across younger and older adults for both information domains.

**Figure 1.**
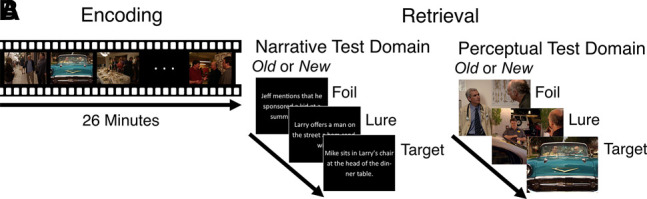
(*A*) Participants viewed a 26-min episode of a sitcom. (*B*) Old/new recognition task based on narrative or perceptual details, with order of test domain counterbalanced across participants. Each recognition task consisted of 30 targets (described or depicted moments from the video encoded), similar lures (moments described or depicted as being similar to the video encoded), and novel foils (described or depicted moments not from the video encoded).

We predicted no differences in basic recognition of repeated targets and novel foils across age groups based on prior MST results (Stark et al. 2013, 2019; Toner et al. 2009; Holden et al. 2013). In line with prior work showing decreased performance in perceptual lures among older adults (Toner et al. 2009; Holden et al. 2013; Stark et al. 2013, 2019), we further predicted greater age-related deficits in perceptual lure discrimination than narrative lure discrimination. Relatively intact memory for narrative details may reflect being able to rely on narrative structure or the meaning of events.

## Results

### Target recognition does not significantly differ across age

**Figure 2.**
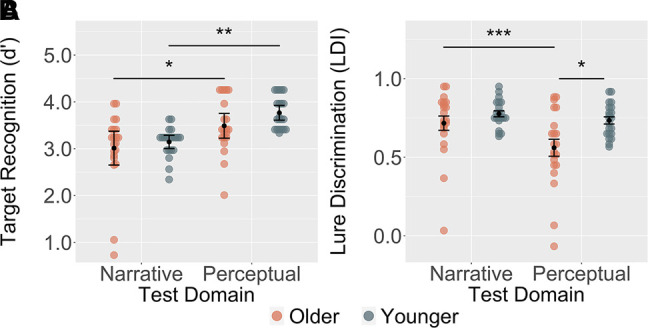
Average performance on target recognition *d*′ and lure discrimination LDI across age groups and test domains. (*A*) Target recognition did not significantly differ across age but differed across test domains. (*B*) Significant age-related differences in perceptual but not narrative lure discrimination. Points represent individual participants’ mean performance. Bars represent average performance (±standard error of the mean). Significant tests: (*) *P* < 0.05, (**) *P* < 0.01, (***) *P* < 0.001.

Target recognition was assessed in terms of normalized *d*′ values derived from signal detection analysis (see the Materials and Methods). To do so, we performed a 2 × 2 ANOVA incorporating a within-subjects factor of test domain (narrative vs. perceptual) and between-subjects factor of age (older vs. younger). The comparison revealed a significant main effect of test domain (*F*_(1,40)_ = 26.70, *P* < 0.001) but not for age (*F*_(1,40)_ = 2.50, *P* = 0.12) (see Fig. 2A). Additionally, no significant interaction was observed (*F*_(1,40)_ = 0.46, *P* = 0.50). Post-hoc contrasts revealed that participants performed significantly better at target recognition for perceptual compared with narrative test domains in both older (*t*_(40)_ = 3.17, *P* = 0.02 corrected) and younger (*t*_(40)_ = 4.14, *P* = 0.001 corrected) age groups. Findings remain the same with the nonnaïve younger participant excluded (see the Materials and Methods), resulting in a significant main effect of test domain (*F*_(1,39)_ = 27.04, *P* < 0.001), no significant main effect of age (*F*_(1,39)_ = 2.58, *P* = 0.12), and no significant test domain × age interaction (*F*_(1,39)_ = 0.63, *P* = 0.43). Although there were no age differences in overall recall performance, additional linear mixed-effects model analyses were conducted to ensure that overall memory ability did not account for recognition performance differences. Linear mixed-effects models with age and test domain as fixed effects and recall performance as a random effect were conducted to account for variability in recall performance. The models revealed a significant effect of test domain (*F*_(1,41.01)_ = 3.09, *P* = 0.004). No significant effect of age (*F*_(1,77.21)_ = 0.85, *P* = 0.40) or interaction (*F*_(1,41.01)_ = 0.63, *P* = 0.53) was observed. In sum, recognition of previously studied information was easier for perceptual compared with narrative details, and this was consistent across age groups. Importantly, basic recognition did not differ as a function of age, even when accounting for overall recall ability.

### Age-related discrimination deficits for perceptual, but not narrative, details

To assess discrimination of similar lure items, we performed a 2 × 2 ANOVA with a within-subjects factor of test domain (narrative vs. perceptual) and between-subjects factor of age (older vs. younger). We corrected for response bias by calculating the lure discrimination index (LDI) for each participant (see the Materials and Methods). The comparison revealed a significant main effect of test domain (*F*_(1,40)_ = 22.16, *P* < 0.001) and age (*F*_(1,40)_ = 5.46, *P* = 0.02), showing that, on average, older adults were poorer at rejecting similar lures than younger adults. Additionally, results show a significant interaction between age and test domain (*F*_(1,40)_ = 7.35, *P* = 0.01), indicating that age group differences in lure rejection rates varied as a function of whether narrative versus perceptual lures were being tested (see Fig. 2B). Pairwise comparisons revealed that the interaction was driven by better discrimination in younger than older adults for perceptual (*t*_(40)_ = 2.96, *P* = 0.03 corrected), but not for narrative (*t*_(40)_ = 1.21, *P* = n.s. corrected), lures. Moreover, older adults showed better performance on narrative compared with perceptual lures (*t*_(40)_ = 5.25, *P* < 0.0001). No other pairwise contrasts were significant. Findings remain the same with the nonnaïve younger participant excluded, resulting in a significant main effect of age (*F*_(1,39)_ = 5.11, *P* = 0.03), test domain (*F*_(1,39)_ = 21.37, *P* < 0.001), and significant test domain × age interaction (*F*_(1,39)_ = 6.84, *P* = 0.01). Unlike basic recognition, rejection of similar lures differed across age groups. This difference was driven by poorer discrimination of perceptual, but not narrative, lures. An additional linear mixed-effects model with age and test domain as fixed effects and recall performance as a random effect was conducted to account for recall variability. When accounting for a recall performance in the model, test domain predicted lure discriminability performance (*F*_(1,36.73)_ = 4.62, *P* < 0.001) rather than age (*F*_(1,76.79)_ = 0.41, *P* = 0.68), suggesting that significant main effect of age may be explained by recall variability. The model still reveals an interaction (*F*_(1,36.73)_ = 2.59, *P* = 0.01), suggesting that differences in lure rejection rates varied across age groups, even when accounting for age and performance on a separate free recall task. Finally, we note that we ran a separate pilot study in a sample of younger adults to ensure comparable difficulty of lures across test domains (see the Materials and Methods for details).

## Discussion

In the present study, we sought to examine age-related changes in recognition memory for narrative and perceptual information. Younger and older participants viewed an episode of a television sitcom and later completed an old/new recognition test consisting of targets, foils, and similar lure items that tapped into perceptual and narrative domains. Critically, to our knowledge, this is the first study to examine mnemonic discrimination of perceptual information in memory alongside specific narrative details. Analyses revealed better performance on basic recognition of repeated targets and novel foils for perceptual compared with narrative trials across age groups. Discrimination of similar lures, however, differed across age groups, with older adults showing a deficit in correctly rejecting perceptual, but not narrative, lures. Importantly, lure discrimination ability was equated across domains in younger adults, suggesting that the perceptual discrimination task is not inherently more difficult. Moreover, older adults performed comparably with younger adults at discriminating highly similar narrative lures from information in the episode.

Our results demonstrate the utility of including measures for more than one type of memory for the same complex stimulus. We adapted a widely used paradigm—the MST—that typically aims to tax pattern separation processes in the hippocampus (Stark et al. 2013, 2019); however, rather than testing solely on perceptual details as previous paradigms have done, we tested detailed recognition of narrative information as well. Memory for narrative details is often tested with spoken or written free recall, which is a different and potentially more taxing form of memory retrieval than cued recognition (Craik and McDowd 1987). Some findings showing age-related deficits in recall may be results affected by the difficulty of the task itself. Additionally, recall tests tend to focus largely on narrative details and lessen the focus on perceptual details. The use of a recognition test in our design allowed us to directly assess differences between perceptual and narrative domains while minimizing age-related differences based on the nature of the task. Thus, our results are driven by differences in the information domain (i.e., perceptual and narrative) rather than the format of the test (e.g., recall vs. recognition), which suggest that perceptual and narrative domains may tax distinct cognitive processes.

It has been argued that aging is associated with a loss of detailed memory but a relative preservation of gist (Schacter et al. 1997). This is often operationalized as retention of central, general features of studied material but loss of specific (sometimes peripheral) information, resulting in either forgetting or false recognition due to interference (Koutstaal and Schacter 1997; Norman and Schacter 1997; Tun et al. 1998). Broadly in line with this work and prior studies using MST variants (Stark et al. 2013, 2015, 2019), we found an increase in false alarms to lures but a relative preservation of target recognition in older adults. This can be viewed as a shift away from detailed memory in aging. Many prior studies suggesting a gist versus detail trade-off have used static images (Stark et al. 2015) or word lists (Norman and Schacter 1997) as stimuli, using false alarms as the key measure. However, continuous events captured by narratives may allow us to tap into distinct mechanisms that go beyond simple visual versus verbal representations. A study by Adams et al. (1997) tested verbal narrative recall of younger and older adults and showed age-related deficits in verbatim details, but that older adults showed a greater tendency toward processing a story's interpretive meaning. Our results may expand on this phenomenon. Specifically, by testing both simple target recognition and lure discrimination (more taxing of detailed memory representations) across perceptual and narrative domains, our findings suggest that older participants may be more able to retain detailed memory for information that relates to a story's meaning.

Alternatively, one potential explanation for the relative deficit in perceptual but not narrative lure discrimination among older adults could be an overall difficulty with visual perception. Although we formally included participants with corrected-to-normal vision and ensured they could see the computer screen well, a caveat of this study is that we did not conduct a formal vision test in the laboratory. While visual acuity is sometimes reported to decrease with age, older adults performed similarly to younger adults on the target recognition assessment. There may also be age-related attentional differences beyond low-level visual perception (Verhaeghen and Cerella 2002; Glisky 2007). For example, older adults may simply have attended to the screen to a lesser degree. Although we did not collect pertinent data in this study (e.g., eye tracking) and cannot speak to this directly, future studies in this vein can assess the role of attention and top-down control.

Our findings are in agreement with studies investigating interference in memory pertaining to visual information, revealing a deficit specifically for lure discriminability but not target recognition (Yassa et al. 2011b; Toner et al. 2013; Stark et al. 2015; Foster and Giovalleno 2020; Chamberlain et al. 2022). Similar to this study, this may drive a poorer ability to pattern separate similar information in older adults. Our findings may expand on this by demonstrating that this specifically targets perceptual, but not narrative, lures. Although we explicitly tested fine-grained details across both domains and took steps to equate task difficulty in younger adults (see the Supplemental Material; Supplemental Table S1), highly detailed memories may be inherently more likely to tap into perceptual representations (Robin and Moscovitch 2017). Moreover, in the context of this study, it is possible that conditions of narrative lure discrimination may rely on gist-based or more semantically driven representations in older adults. Thus, to some extent, our findings may reflect age-related differences in processing gist versus detailed information with age. In line with this, it has been recently argued that an age-related shift from detailed to gist representations may be driven by multiple factors beyond cognitive decline, including changes in priorities and goals associated with aging (Grilli and Sheldon 2022).

Importantly, the cognitive processes targeted by our study may rely on differentially vulnerable neural mechanisms in the aging brain. Memory representations extend beyond the hippocampus into larger cortico–hippocampal networks, which may differ based on information type. According to one well-supported view, content in memory is dissociated into a posterior–medial (PM) system that supports spatiotemporal, contextual, and situational details and an anterior–temporal (AT) system that tracks items, objects, and individual people (Ranganath and Ritchey 2012; Ritchey et al. 2015; Reagh and Ranganath 2018). In this framework, the PM system would more preferentially support narrative details, whereas the AT network may support more perceptually driven information. Given that narrative structure, mediated by the PM network, provides a way to deeply encode information by allowing us to bridge overarching themes and create meaningful associations, we anticipated that older participants would perform better at recognizing narrative details aided by these associative anchors. However, differences in basic recognition performance based on test domain were driven by better (not worse) performance on perceptual information. Importantly, this effect was present across age groups, suggesting that there may be other reasons such as visual salience or difficulty level across domains that underlie this result in terms of basic recognition memory. Critically, age-related discrimination deficits were limited to perceptual lures despite perceptual target recognition performance being better across both groups than narrative recognition. This further suggests that the selective deficits observed in older adults at perceptual, but not narrative, lure discrimination did not arise as a mere function of task demands differing.

Although this study did not examine age-related pathology, this pattern of results may provide insights into the integrity of the aging brain. Increasing evidence suggests that PM and AT systems are differentially vulnerable to age-related pathology. Accumulation of tau is associated with impairment of episodic memory processes and is strongly predictive of Alzheimer's disease. Early stages of Alzheimer's disease are thought to originate in AT regions, as tau depositions accumulates in these areas (Braak and Braak 1997). Increased tau depositions coupled with amyloid plaques later spread in the PM regions, resulting in the progression of Alzheimer's disease (Jagust 2018; Leal et al. 2018). Our results are in line with other findings suggesting that AT-mediated processes may be more generally vulnerable in aging (Reagh et al. 2016, 2018). Together, findings of this sort suggest an increasing vulnerability of PM-mediated processes in aging, perhaps especially in Alzheimer's disease. Although our sample does not include formally diagnosed dementia patients, our study may provide insights into future studies related to Alzheimer's disease. Exploratory analyses that incorporated a contrast of cognitive ability indicate that declines in perceptual lure discrimination were largely driven by older adults with poorer global cognitive ability (see the Supplemental Material; Supplemental Fig. S2A,B). Future work can examine this in more detail.

In sum, our study used a mnemonic similarity task applied to a naturalistic stimulus to show age-related deficits in perceptual, but not narrative, lure discrimination. In line with several existing studies, we found domain-selective recognition deficits as a function of aging (Reagh et al. 2016, 2018; Güsten et al. 2021). These data indicate that domain selectivity of age-related memory deficits extends to memory for continuous, lifelike information beyond simple laboratory experiments. Perceptual details, which are not anchored by narrative associations, may be particularly vulnerable in the context of aging. Additionally, our findings suggest that cognitive decline may amplify lure discrimination deficits. Testing memory for different aspects of experiences may offer important insights into memory ability in healthy and pathological aging, and a naturalistic approach offers us insights into how these processes operate in real-world situations.

## Materials and Methods

### Participants

Forty-two participants were recruited from the Davis, California, community: 21 younger adults (*M* = 20.04, SD = 1.81; range = 18–25; 20 female) and 21 older adults (*M* = 73, SD = 7.43; range = 61–93; 10 female). The study was approved by the Institutional Review Board of University of California, Davis, and all participants provided written consent before participating in the study. Younger adults were recruited from a pool of undergraduate students enrolled in psychology courses at the University of California. Inclusion criteria for younger adults included normal hearing, normal or corrected-to-normal vision, no history of major neurological or psychiatric illness, and English as a native language. Older adults were recruited from the Davis community through online advertisement, flyers, and word of mouth. Older participants were initially contacted by phone or e-mail for a prescreening interview. Inclusion criteria for older adults were the same as for younger adults, except the requirement of English as a native language was relaxed to include individuals who began fluency in English before age 5. All participants were naïve to the stimulus, with the exception of one younger participant (i.e., reported having seen *Curb Your Enthusiasm* prior to the study). Results remain the same even after the exclusion of the nonnaïve younger participant (see the Results). No older adults recruited for the study had formal diagnoses of cognitive or neurological disorders, including dementia or mild cognitive impairment. However, a portion of our older adult sample exhibited scores on neuropsychological tests below standardized cutoffs, which we leveraged for exploratory analyses (see the Supplemental Material; Supplemental Tables S2, S3).

### Materials, design, and procedure

Older participants completed the following neuropsychological tests to assess for cognitive impairments: Craft21 recall immediate, Craft21 recall delayed, Montreal Cognitive Assessment (MoCA), and Multilingual Naming Test (MINT) (see Table 1). Briefly, Craft21 assesses recall for narratives, MoCA coarsely assesses cognitive ability, and MINT assesses the ability to name objects in English. Older and younger participants viewed a 26-min episode of a television show (HBO's *Curb Your Enthusiasm*, S01E07: “AAMCO”) and then completed a free recall task, a recognition task, and an event segmentation perception task (not included here). For the recall task, participants were instructed to recall everything that they could remember about the episode in as much detail as possible. Manually scored recall (Levine et al. 2002) resulted in no age-related differences in overall recall performance (see Supplemental Table S2). The present analyses mainly focus on recognition memory task performance.

**Table 1.**
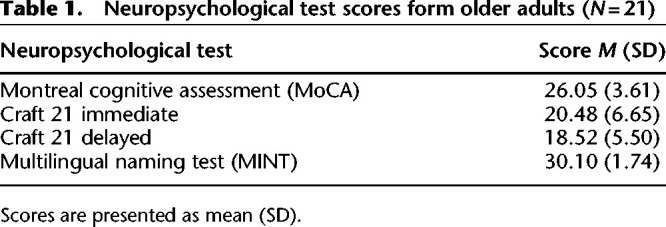
Neuropsychological test scores form older adults (*N* = 21)

Participants completed two recognition tasks based on narrative or perceptual details, wherein the narrative recognition task consisted of identifying sentences as old or new via button press, and the perceptual recognition task consisted of identifying images as old or new via button press. We aimed to test recognition memory for highly specific information by adapting a mnemonic similarity task approach. Briefly, in addition to old/new recognition, this recognition task variant includes similar lure trials that induce mnemonic interference. Critically, sentences and images were either studied targets (described or depicted moments from the video encoded), similar lures (moments described or depicted as being similar to the video encoded but differing subtly from the video encoded), and novel foils (described or depicted moments clearly not from the video encoded). An example a lure in the narrative test domain is “Larry offers a man on the street a ham sandwich” when the correct answer is “Larry offers a man on the street a tuna sandwich” (see Supplemental Fig. S1A). Similarly, an example of a lure of perceptual test domain is an image of Larry at a similar auto shop from a different episode (S01E08) (see Supplemental Fig. S1B). An example of narrative and perceptual test domain includes plausible descriptions or depicted moments such as “Larry goes to see Dr. John Lynch on the third floor of the medical building” (S11E04). Each recognition task consisted of 30 targets, 30 lures, and 30 foils. The order of narrative and perceptual recognition tasks was counterbalanced and pseudorandomized such that odd-numbered participants completed the narrative recognition task first followed by the perceptual recognition task, and even-numbered participants completed the perceptual recognition task first followed by the narrative recognition task.

A key step in comparing task conditions across age groups is to ensure that those conditions do not merely reflect differences in difficulty. To address this, we gathered ratings for each test stimulus from a sample of younger adult participants. Twenty-three participants (*M* = 20.14, SD = 0.94; range = 18–22; 14 female) watched the television episode used in the main study and were later shown a series of descriptions and images. For each target, foil, and lure trial, participants rated the difficulty of correctly accepting or rejecting each image or description on a scale of 1–5. In addition to rating the difficulty, participants were notified that lure images or descriptions were not from the encoded video and were instructed to rate its similarity to the encoded video. Difficulty and similarity ratings for narrative and perceptual domain and trial type were not statistically different (see Table 2; Supplemental Material). Although we cannot completely rule out differences in difficulty, this pilot sample indicates that the narrative and perceptual test domains are comparably challenging in younger participants.

**Table 2.**
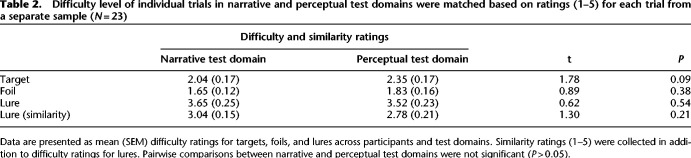
Difficulty level of individual trials in narrative and perceptual test domains were matched based on ratings (1–5) for each trial from a separate sample (*N* = 23)

### Analyses

Mean proportion of correct responses for each trial type were calculated (see Table 3). Recognition performance was scored as the proportion of targets, lures, and foils endorsed as being new or old. Targets were scored as hits if endorsed as old and as misses if endorsed as new. Lures and foils were endorsed as correct rejections as new and as false alarms if endorsed as old. Additionally, target recognition was assessed in terms of *d*′ values (*z*[target hit rate] − *z*[foil false alarm rate]) derived from signal detection analysis. Older and younger adults’ recognition performance were compared using pairwise independent sample *t*-tests within each trial type. Additionally, we calculated a lure discrimination index (LDI) (Stark et al. 2013, 2019) for each subject (*p*[new|lure] − *p*[old|foil]). Data were analyzed using repeated measures ANOVAs, and post-hoc contrasts were corrected for multiple comparisons using the Bonferroni method. Although there were no age differences in overall recall performance, additional linear mixed-effects model analyses were conducted to ensure that overall memory ability did not account for recognition performance differences. Recall performance was entered as a random covariate into a linear mixed-effects model predicting target recognition performance [*d*′ ∼ age group × test domain + (1|recall performance)] and lure discriminability performance [LDI ∼ age group × test domain + (1|recall performance)]. Statistical analysis was performed in R (version 4.0.3, https://www.r-project.org) using the afex package (https://github.com/singmann/afex).

**Table 3.**
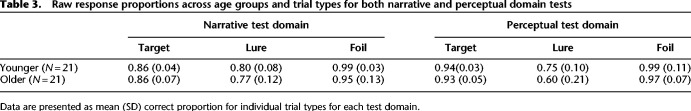
Raw response proportions across age groups and trial types for both narrative and perceptual domain tests

### Data Deposition

The full stimuli for the materials used in the present experiment, anonymized data files, coded data, R Markdown files, and Jupyter Notebook files containing the analysis scripts are available on Open Science Framework (https://osf.io/3qe9w) and GitHub (https://github.com/aidelarazan/curbage_recognition).

### Competing interest statement

The authors declare no competing interests.

## Supplementary Material

Supplemental Material
