# Supplementary material for: Aging impacts memory for perceptual, but not narrative, event details
Source: Learn Mem. 2023 Feb;30(2):48–54. doi: 10.1101/lm.053740.122 (PMC9987157; doi:10.1101/lm.053740.122)
Supplement: Supplemental Material [file supp_30_2_48__DC1.html]

Supplemental Material 

# Aging impacts memory for perceptual, but not narrative, event details

## Supplemental Material

- Supplemental\_Material\_.pdf
