# Supplementary material for: Aging impacts memory for perceptual, but not narrative, event details
Source: Learn Mem. 2023 Feb;30(2):48–54. doi: 10.1101/lm.053740.122 (PMC9987157; doi:10.1101/lm.053740.122)
Supplement: Supplemental Material [file supp_30.2.48_Supplemental_Material_.pdf]

Supplemental Materials

Aging impacts memory for perceptual, but not narrative, event details

Angelique I. Delarazan, Charan Ranganath, and Zachariah M. Reagh
Department of Psychological & Brain Sciences, Washington University in St. Louis
Center for Neuroscience, University of California, Davis
*Corresponding author: a.delarazan@wustl.edu

DATA CODE AND AVAILABILTY

The full stimuli for the materials used in the present experiment, anonymized data files, coded data, R Markdown, and Jupyter Notebook files containing the analysis scripts are available on Open Science Framework (https://osf.io/3qe9w/) and GitHub (https://github.com/aidelarazan/curbage_recognition).

MATERIALS, DESIGN, AND PROCEDURE

Matched task difficulty and subjective lure similarity of Narrative and Perceptual test domains

Recognition tasks based on narrative or perceptual details consisted of sentences or images that were studied targets, similar lures, and novel foils. Targets described or depicted moments from the video encoded (S01E07). Lures of Narrative test domain subtly changed information or details (e.g., "Larry offers a man on the street a *ham* sandwich" when the correct answer is "Larry offers a man on the street a *tuna* sandwich", Supplemental Figure 1A). Similarly, lures of perceptual test domain consisted of images from that were similar to the video encoded (e.g., Larry at a car repair shop; Supplemental Figure 1B). Foils of Narrative and Perceptual test domain described or depicted moments from a different episode that was not encoded in the experiment (e.g., "Larry goes to see Dr. John Lynch on the third floor of the medical building;" S11E04).

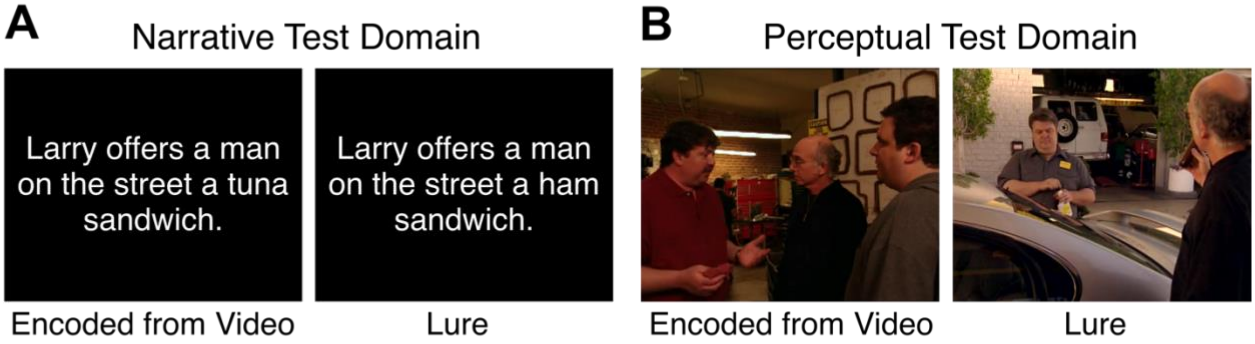

Supplemental Figure 1. Example of lure trials for (A) Narrative and (B) Perceptual test domains. Lures (right) depict sentences or image that are similar to the video encoded (left) but differed subtly from the video encoded. In the Narrative test domain example, a detail about a particular item from the scene is altered. In the Perceptual test domain example, a similar scene at a different auto shop is depicted.

Difficulty level of individual trials in narrative and perceptual domains were matched based on ratings for each trial from a separate pilot sample of twenty-three younger adult participants ($M = 20.14$, $SD = 0.94$; range = 18 - 22; 14 female). Participants watched the episode and were later shown a series of descriptions and images. For each target, foil, and lure trials, participants rated the difficulty of correctly accepting or rejecting each image or description on a scale of 1-5. In addition to rating the difficulty, participants were notified that lure images or descriptions were not from the encoded video and were instructed to rate its similarity to the encoded video. Difficulty and similarity ratings for Narrative and Perceptual domain and trial type were not statistically different (see Table 2). Separate pairwise comparisons between Narrative and Perceptual domains revealed no differences in difficulty in targets ($t(22) = 1.78$, $p = .09$), foils ($t(22) = 0.89$, $p = .38$), and lures ($t(22) = 0.62$, $p = .54$). Additionally, pairwise comparisons between Narrative and Perceptual domains revealed no difference in similarity ratings in lures ($t(22) = 1.30$, $p = .21$). Findings from our pilot sample ensured matched difficulty level across test domains, as normalized to younger adult performance.

No age-related differences in overall recall of verifiable details

Recall performance was quantified using a manual scoring method adapted from the Autobiographical Interview (Levine et al., 2002). Two raters segmented each recall transcript into meaningful detail units and then assigned labels that describe the content (e.g., Verifiable Details, Unverifiable Details, etc.). Each rater scored the number of verifiable details each participant recalled, and the final recall score resulted is the average score from the two raters. Pairwise comparison between age group revealed no differences in the number of verifiable details recalled ($t(30.01) = 1.46$, $p = .16$; Supplemental Table S1).

Supplemental Table 1. Total average verifiable details in recall for younger and older adults. Data are presented as mean (SD) across participants.

	**Total Verifiable Details in Recall**		
	M(SD)	t	p
Younger$_{(N=21)}$	172.90 (102.19)	1.46	0.16
Older$_{(N=21)}$	134.69 (58.71)		

RESULTS

Narrative recognition correlates with neuropsychological tests of story recall

A series of neuropsychological tests was collected to assess for cognitive impairments among older adults (see Supplemental Table S2). To determine whether variation in recognition performance on our two tasks could be explained by performance on standard neuropsychological tests, we conducted a series of correlations (Pearson's) between participants' individual neuropsychological scores and participants' d' measure on Narrative and Perceptual test domains. We observed a significant positive association between d' for Craft 21 Delayed neuropsychological test and Narrative test domain ($r_{(19)} = 0.46$, $p = .04$) and Perceptual test domain ($r_{(19)} = 0.46$, $p = .04$). This may reflect that both the Craft 21 test and our video stimulus hinged on understanding of a story. Additionally, we conducted correlation between neuropsychological test and participants' LDI scores. Results showed a significant positive correlation between LDI for the Narrative test domain and the Craft 21 Story Delayed ($r_{(19)} = 0.50$, $p = .02$). No other correlations with LDI were statistically significant. These correlations between the Craft 21 recall test and Narrative recognition and discrimination may reflect a common demand on narrative comprehension.

*Supplemental Table 2. Neuropsychological test scores for older adults, and correlations between these scores and recognition performance on Narrative and Perceptual test domain. Scores are presented as mean (SD) for neuropsychological tests. Correlations (r) and significance are displayed between average score and target recognition (d') and lure discrimination (LDI) for each domain. Significant tests: *$p < 0.05$*

**Neuropsychological Test**		**Narrative Test Domain**				**Perceptual Test Domain**			
		d'		*LDI*		*d'*		*LDI*	
	M(SD)	r	p	r	p	r	p	r	p
MoCA	*26.05 (3.61)*	-0.13	0.58	0.19	0.41	0.03	0.91	0.41	0.07
Craft 21 Immediate	*20.48 (6.65)*	0.34	0.13	0.37	0.10	0.38	0.09	0.21	0.35
Craft 21 Delayed	*18.52 (5.50)*	0.46	0.04*	0.50	0.02*	0.46	0.04*	0.36	0.11
MINT	*30.10 (1.74)*	0.12	0.61	0.21	0.37	-0.17	0.45	0.07	0.77

Perceptual discrimination deficits as a function of MoCA scores

Neuropsychological tests aim to assess cognitive impairments and are often administered to older adults. Of the neuropsychological tests collected, the MoCA provides the most comprehensive test that coarsely assess for multiple cognitive processes including: short-term memory, visuospatial abilities, executive functions, attention, working memory, language and orientation to time and place. The MoCA is well-validated in testing older adults for mild cognitive impairment. MoCA scores range from 0-30, and a score of 26 or higher is generally considered "normal" (Nasreddine et al., 2005). Our older adult sample exhibited heterogeneity in falling above or below a MoCA score of 26. In line with prior studies, we next incorporated a simple contrast of cognitive ability in our analyses via split based on MoCA score (Holden et al. 2013; Pishdadian et al., 2020). Older participants were categorized in two groups: Low MoCA Scorers (MoCA score < 26, $N = 8$) and High MoCA Scorers (MoCA score ≥ 26, $N = 13$), and similar comparisons between age group (Older vs. Younger) and test domain (Narrative vs. Perceptual) on d' and LDI measures were conducted. Thus, although we did not plan to split older adults by neuropsychological tests a priori, we leveraged this variability in our older adult sample to conduct additional analyses. Mean proportion of

correct responses for each trial type were calculated (Supplemental Table S3). Analyses reported previously were repeated (see Methods) incorporating this subgroup split in the older adult sample.

Supplemental Table 3. Raw response proportions for older adults with Low (MoCA Score < 26) and High (MoCA Score ≥ 26) MoCA Scores and younger adults. Data are presented as mean (SD) proportion correct responses.

	**Narrative Test Domain**			**Perceptual Test Domain**		
	Target	Lure	Foil	Target	Lure	Foil
Younger	0.86 (0.04)	0.80 (0.08)	0.99 (0.03)	0.94 (0.03)	0.75 (0.10)	0.99 (0.11)
Older$_{\text{(Low MoCA Score)}}$	0.88 (0.08)	0.72 (0.11)	0.96 (0.12)	0.93 (0.05)	0.53 (0.18)	0.93 (0.10)
Older$_{\text{(High MoCA Score)}}$	0.84 (0.07)	0.81 (0.12)	0.95 (0.14)	0.93 (0.04)	0.65 (0.22)	0.99 (0.03)

High, not low, MoCA scorers perform similarly to younger adults in target recognition

We performed a 2 x 3 ANOVA with test domain (Narrative vs. Perceptual) and group (Older$_{\text{High MoCA Scorer}}$ vs. Older$_{\text{Low MoCA Scorer}}$ vs. Younger) based on MoCA scores to determine whether there are differences in target recognition for older participants with low or high MoCA scores. Results reveal a significant main effect of test domain ($F(1, 39) = 18.68$, $p < .001$) and trending interaction ($F(2, 39) = 2.77$, $p = .075$; Supplemental Figure 2A). Pairwise comparisons revealed that these effects were qualified by better performance on Perceptual compared to Narrative items for younger ($t(39) = 4.34$, $p = .002$ corrected) and older participants with high MoCA scores ($t(39) = 4.00$, $p = .004$), but not older participants with low MoCA scores ($t(39) = 0.29$, p = n.s. corrected). This shows that high MoCA older participants behaved similarly to younger participants, performing better at target recognition for Narrative but not Perceptual test domains. This difference was not observed in older participants with low MoCA scores.

Perceptual discrimination deficits are driven by older adults with low MoCA scores

To determine whether there are differences in discrimination of similar lure items among older participants who scored high and low on the MoCA, we conducted similar analyses as above with LDI. Results show a significant main effect of group ($F(2, 39) = 4.52$, $p = .017$), a significant main effect of test domain ($F(1, 39) = 31.12$, $p < .001$), and a significant group X test domain interaction ($F(2, 39) = 4.82$, p =

.014) for comparisons between younger, older participants with low MoCA scores, and older participants with high MoCA scores (Supplemental Figure 2B). Post-hoc pairwise comparisons revealed that the interaction was driven by poorer discrimination of similar lure items of Perceptual test domain among older adults with low MoCA scores ($t(39) = 4.42$, $p = .001$ corrected) and older adults with high MoCA scores ($t(39) = 3.29$, $p = .03$). No pairwise comparisons for test domain was significant among younger adults ($t(39) = 1.43$, p = n.s.). Additional pairwise comparisons revealed a significant difference between younger and older low MoCA scorers ($t(39) = 3.69$, $p = .01$), but not between younger and older high MoCA scorers ($t(39) = 1.68$, p = n.s.) in Perceptual test domain. Together, these results suggest that the disproportionate deficits in perceptual lure discrimination exhibited by older adults could have been driven by the subset of older adults showing evidence of poorer global cognitive functioning.

Exploratory analyses above divided older adults based on low versus high performance on the MoCA, allowing us to incorporate a contrast of global cognitive ability. These analyses indicated no significant differences in recognition performance between younger and older adults with high cognitive abilities, whereas better recognition performance on perceptual compared to narrative domain was not observed in older adults with low cognitive abilities. Furthermore, age-related deficits in lure discrimination performance on perceptual domains was mostly driven by older adults showing evidence for poorer overall cognitive ability. Though this study did not examine age-related pathology, this pattern of results may provide insights into the aging brain in the context of vulnerability to further decline.

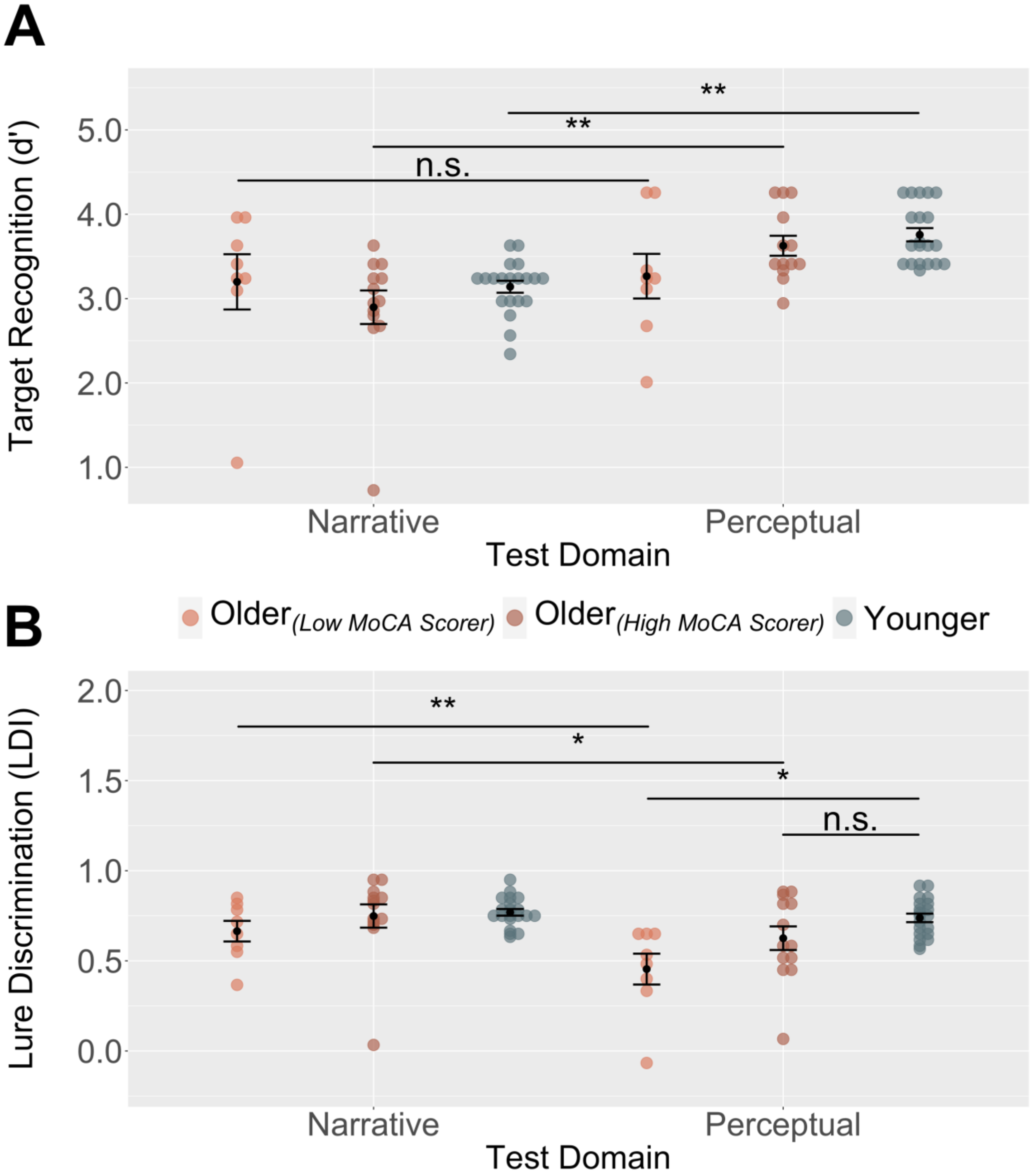

Supplemental Figure 2. Average performance on target recognition (d') and lure discrimination (LDI) across age group and test domain after split on neuropsychological test performance. Older adult sample divided into Low and High MoCA scorers, falling below or above "normal" score of 26. (A) No significant differences in test domain for target recognition in both High MoCA Score older adults and younger adults, but significant difference for Low MoCA Scorer older adults. (B) Significant differences in Perceptual lure discrimination were observed between young and older adults with Low but not High MoCA scores. No significant differences were observed for Narrative lure discrimination. Key: Points

represent individual participants' mean performance. Bars represent average performance (+/- standard error of the mean). Significant tests: * $p < .05$, ** $p < .01$, *** $p < .001$
